# Importance-Performance Analysis of Personal Health Records in Taiwan: A Web-Based Survey

**DOI:** 10.2196/jmir.7065

**Published:** 2017-04-27

**Authors:** Hsiao-Hsien Rau, Yi-Syuan Wu, Chi-Ming Chu, Fu-Chung Wang, Min-Huei Hsu, Chi-Wen Chang, Kang-Hua Chen, Yen-Liang Lee, Senyeong Kao, Yu-Lung Chiu, Hsyien-Chia Wen, Anis Fuad, Chien-Yeh Hsu, Hung-Wen Chiu

**Affiliations:** ^1^ Graduate Institute of Biomedical Informatics Taipei Medical University Taipei Taiwan; ^2^ Administration Department Healthconn Corp Taipei Taiwan; ^3^ Institute of Medical Informatics Department of Computer Science of Information Engineering National Cheng Kung University Tainan Taiwan; ^4^ School Of Public Health National Defence Medical Center Taipei Taiwan; ^5^ National Health Insurance Administration Ministry of Health and Welfare Taipei Taiwan; ^6^ School of Nursing College of Medicine Chang Gung University Linkou Taiwan; ^7^ Division of Endocrinology Department of Pediatrics Linkou Chang Gung Memorial Hospital Linkou Taiwan; ^8^ School of Nursing College of Medicine Chang Gung University Taipei Taiwan; ^9^ Internet of Things Laboratory Chunghwa Telecom Laboratories TaoYuen Taiwan; ^10^ School of Health Care Administration Taipei Medical University Taipei Taiwan; ^11^ Department of Biostatistics, Epidemiology and Population Health Faculty of Medicine Universitas Gadjah Mada Yogyakarta Indonesia; ^12^ Department of Information Management National Taipei University of Nursing and Health Sciences Taipei Taiwan; ^13^ Master Program in Global Health and Development Taipei Medical University Taipei Taiwan

**Keywords:** electronic health records, health records, personal, surveys and questionnaires

## Abstract

**Background:**

Empowering personal health records (PHRs) provides basic human right, awareness, and intention for health promotion. As health care delivery changes toward patient-centered services, PHRs become an indispensable platform for consumers and providers. Recently, the government introduced “My health bank,” a Web-based electronic medical records (EMRs) repository for consumers. However, it is not yet a PHR. To date, we do not have a platform that can let patients manage their own PHR.

**Objective:**

This study creates a vision of a value-added platform for personal health data analysis and manages their health record based on the contents of the "My health bank." This study aimed to examine consumer expectation regarding PHR, using the importance-performance analysis. The purpose of this study was to explore consumer perception regarding this type of a platform: it would try to identify the key success factors and important aspects by using the importance-performance analysis, and give some suggestions for future development based on it.

**Methods:**

This is a cross-sectional study conducted in Taiwan. Web-based invitation to participate in this study was distributed through Facebook. Respondents were asked to watch an introductory movie regarding PHR before filling in the questionnaire. The questionnaire was focused on 2 aspects, including (1) system functions, and (2) system design and security and privacy. The questionnaire would employ 12 and 7 questions respectively. The questionnaire was designed following 5-points Likert scale ranging from 1 (“disagree strongly”) to 5 (“Agree strongly”). Afterwards, the questionnaire data was sorted using IBM SPSS Statistics 21 for descriptive statistics and the importance-performance analysis.

**Results:**

This research received 350 valid questionnaires. Most respondents were female (219 of 350 participants, 62.6%), 21-30 years old (238 of 350 participants, 68.0%), with a university degree (228 of 350 participants, 65.1%). They were still students (195 out of 350 participants, 56.6%), with a monthly income of less than NT $30,000 (230 of 350 participants, 65.7%), and living in the North Taiwan (236 of 350 participants, 67.4%), with a good self-identified health status (171 of 350 participants, 48.9%). After performing the importance-performance analysis, we found the following: (1) instead of complex functions, people just want to have a platform that can let them integrate and manage their medical visit, health examination, and life behavior records; (2) they do not care whether their PHR is shared with others; and (3) most of the participants think the system security design is not important, but they also do not feel satisfied with the current security design.

**Conclusions:**

Overall, the issues receiving the most user attention were the system functions, circulation, integrity, ease of use, and continuity of the PHRs, data security, and privacy protection.

## Introduction

A personal health record (PHR) is an electronic record of health-related information on an individual that conforms to nationally recognized interoperability standards, and that can be drawn from multiple sources while being managed, shared, and controlled by the individual [1]. PHR includes medical records, lab results, physical assessments, medical history, medication history, physiologic measurements, dietary records, exercise records, and so on. These data can be updated by the user, measured by the measurement equipment automatically uploaded, or by hospitals or clinics that allow data import [2]. The purpose of PHR is to integrate patient health information from a variety of sources, including all patient records, and allow authorized persons to access these records any time and at any place.

PHR is that part of the electronic medical records (EMRs) or Electronic Health Records (EHR) that an individual “owns” and controls. In Taiwan, hospitals adopted EMR in 2004. It was related with the introduction of a basic format of EMR. In 2007, the National Health Informatics Project (NHIP) was promoted to implement the infrastructure of health information by the government to prepare the EMR exchange, and encourage hospitals to use EMR. In 2008, clinical document architecture (CDA) was adopted by the government to create 108 basic formats of EMR. The government of Taiwan also provided incentives to hospitals that adopted EMR. Until 2015, 406 hospitals (90% of Taiwan’s hospitals) already earned rewards and could exchange EMR with each other.

The research on PHR has increased. In the past, EMRs were stored in a large database; different medical service providers in the hospital could access the medical records in the database, but transfer between different hospitals was a problem. Thus, the implementation of PHRs allowed patients to achieve the integration of their PHRs and medical records [3]. Traditionally, most people use paper to write their records, whereas PHRs allow people to easily record and maintain their own health information [4]. With the increasing number of people using EMRs, PHRs were also managed and authorized to be shared through a network in some foreign countries. This approach was found to improve the medical satisfaction of performance, and reduce the cost of medical care with better quality [5].

Some studies listed the benefits of using PHR, including reducing health care costs, improving personal health outcomes, and improving the experience of care for patients and their families [6-10].

The current mechanism of medical record exchange in an EMR exchange center plays a role of a personal EMR platform. Through the Exchange Center, a medical record previously created in any hospital can be accessed from another hospital to reduce duplicate examinations, accelerate diagnosis and treatment, and reduce the medical resource burden. The medication record in the EMR is very conducive to decision-making by doctors to avoid drug allergies and other medical disputes. The importance of a personal EMR platform to a medical institution and its members is like the importance of the clients’ information to an enterprise, which is the basis for providing good service.

Although EMR is already being used well, the medical records are still owned by hospitals; people cannot own and manage their health care record. Therefore, the Ministry of Health and Welfare introduced the concept of “My health bank” in 2015 to let patients own their PHR. In the last three years, this initiative permitted people to download the integrated medical record from the Internet, which includes outpatient and hospital records, diagnosis, drug use records, cost, laboratory test and health examination report, allergies, and so on. It lets people view and manage their health care record at any time and from any place. However, “My health bank” still has some problems. The information it includes is a general report with no detailed content; it doesn’t have medical images, the content is still not standardized, and people can not add their own data regarding diet, exercise, nutrition records, or the health examination report from another examination center.

We still do not have a platform that can let patient manage their own PHR. This study creates a vision of a value-added platform for personal health data analysis and management of health records on the basis of the contents of the "My health bank"; patients can retrieve their health records and medical records through the personal EMR platform to manage their own health conditions. The availability of medical records can contribute to the transparency of the medical records and facilitate immediate access to the medical contents, thereby allowing discussions of disease conditions with the patients’ relatives and friends and related medical personnel. Thus, the ownership of the medical record is reverted to the patient.

The National Committee on Vital and Health Statistics found that a critical success factor for PHRs is the provision of software tools that help patients manage their own health conditions [11].

This research gives a vision of the electronic PHR management platform and uses importance-performance analysis (IPA) to identify the important factors from patients' perspectives. The purpose of this study was to explore consumer perception regarding this kind of platform, and try to both identify the key success factors and important aspects using importance-performance analysis, and give some suggestions for future development based on the findings.

## Methods

### Study Design

In this study, we used a cross-sectional study design. We let the participants watch a Web-based video on the vision of personal EMR platform. After the video introduction, we asked them to fill a structured questionnaire on the Internet (created by Google Form) for quantization of their acceptance and importance of the personal EMR platform’s functions and security.

**Figure 1:**
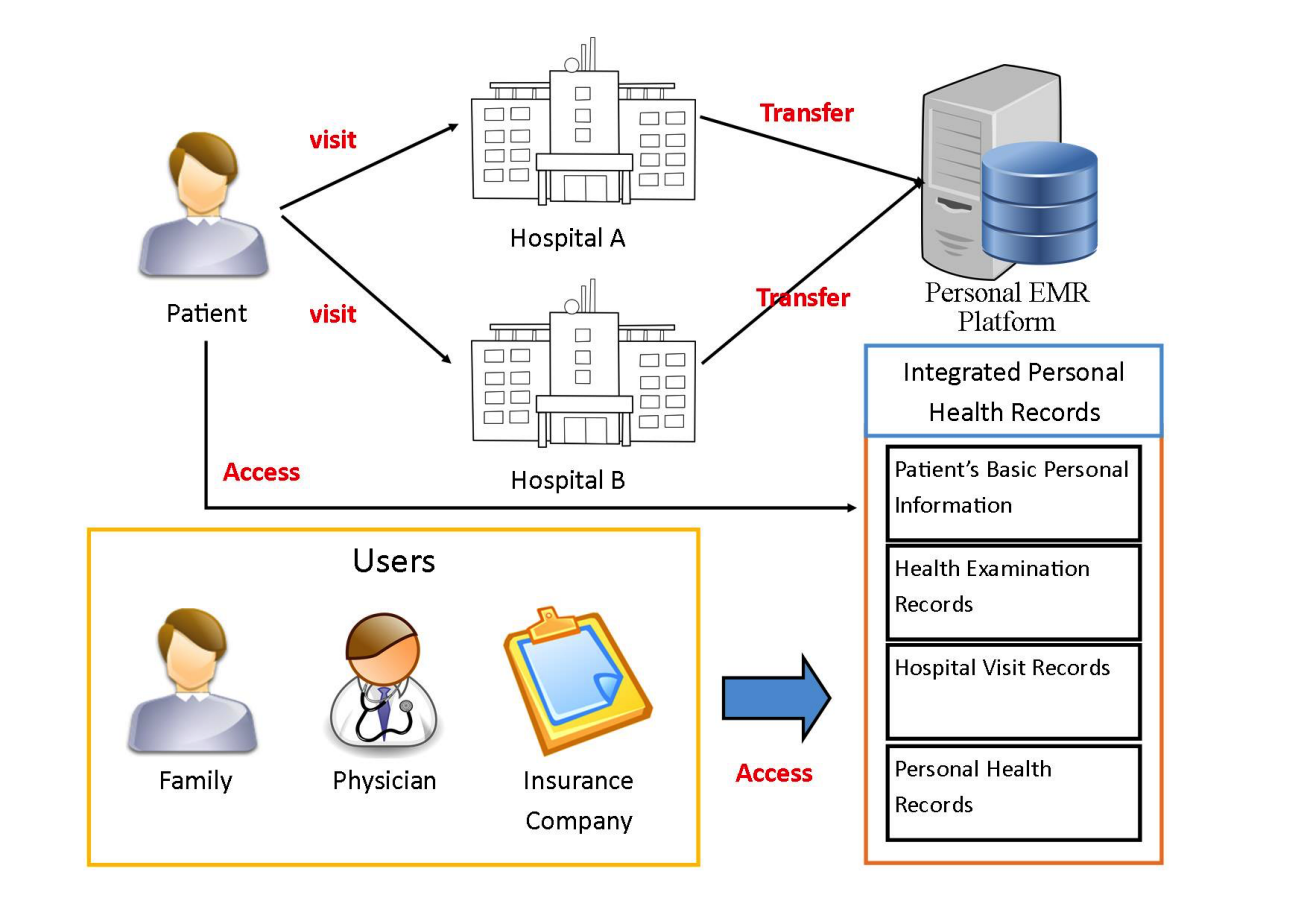
Scenario of personal electronic medical record platform.

In this study, we developed a Web-based video simulation describing the scenario of the personal EMR platform's operation, as shown in Figure 1. This 2:17-min video presented the functions of the personal EMR platform using a case of a patient seeking medical treatment in hospital A. Through the personal EMR platform, after returning home, the patient could use or manage in a secure and private home environment their continuous and integrated PHR, which include personal general information, their health examination record, hospital visit record, PHR, and so on. They can also let doctors in hospital B, family members, and insurance companies use these files through the sharing mechanism when needed.

After viewing the video, the participants were asked to fill out a Web-based questionnaire in Google Form regarding the importance and satisfaction of the performance of the personal EMR platform.

### Questionnaire Design

In order to explore their impact on public acceptance of the personal EMR platform and its importance, this study utilized a questionnaire on importance-performance analysis [12]. The original version of this questionnaire has been used for marketing research to understand consumer perception regarding satisfaction and importance of a product.

We modified this questionnaire into 2 main constructs including (1) system functions and (2) system design and security and privacy, with 12 and 7 questions, respectively. The questionnaire was designed following 5-point Likert scale with the scale ranging from 1 (“disagree strongly”) to 5 (“Agree strongly”).

Before the study, this study invited 4 experts to do expert validity and was pretested by 30 patients. No major problems emerged during this pretest. As showed in Table 1, the questionnaire is highly reliable. The Cronbach alpha of these 2 parts were both more than .9. It means that this questionnaire has a good consistency, and that the subjects could understand the question and fill it in clearly.

**Table 1:** Questionnaire design and reliability analysis.

Aspect	Definition	Questions	Cronbach alpha
System functions	People’s opinion of satisfaction and importance of personal EMR^a^ platform’s system function	12	.936
System design and security	People’s opinion of satisfaction and importance of personal EMR platform’s system design and security	7	.917

^a^EMR: electronic medical record.

### Sampling and Exclusion Criteria

This study used random sampling of the Internet users. We put the video on the YouTube platform from 31st March 2014 to 9th April and publicized it through email and Facebook. After participants finished watching this video, they were asked to fill the questionnaire (Web-based questionnaire in Google sheet format).

The sample size was calculated by using the Magnani [13] formula. It was calculated by reliability coefficient, population proportion (parameter), and width (CI) / margin of error. After calculation, we got the minimum sample size of 323, which meant we would have at least 323 valid respondents after the announcement for 10 days. In total, 614 people have watched this video and 400 people finished the questionnaire survey (response rate: 65.1%); after excluding incomplete and repeated questionnaires, we got 350 valid respondents, which is more than the minimum required case number.

### Data Analysis

This study used Excel (Microsoft) and SPSS (IBM Corp) as statistic tools for analysis. We used descriptive statistics to calculate the mean, standard deviation, median, frequency distribution, and percentage statistics to ascertain the data distribution in system functions, and system design and security. The objective was to examine the relationship between characteristics of participants, as well as all of the aspects above, and set 0.05 as the significance level; if *P*<.05, then it is statistically significant.

We also performed IPA to measure the participants’ attitudes toward the personal EMR platform’s functions. We calculated the mean of every factor in “system functions” and “system design and security” aspects, and we put them in a quadrant diagram.

## Results

### Respondent Characteristics

This research gathered 350 valid questionnaires. As shown in Table 2, most respondents were female (n=219), 21-30 years old (n=238), with a university degree (n=228). Or they were still students (n=195), with a monthly income of less than NT $30,000 (n=230), and lived in the north area (n=236), with a good self-identified health status (n=171).

**Table 2:** Characteristics of respondents.

Characteristics		n (%)
**Gender**		
	Male	131 (37.4)
	Female	219 (62.6)
**Age (years)**				
	<20	46 (13.1)
	21-30	238 (68.0)
	31-40	56 (16.0)
	>40	10 (2.9)
**Highest education level**				
	High school degree	8 (2.3)
	University degree	228 (65.1)
	Institute or above	114 (32.6)
**Employment**		
	Students	195 (55.7)
	Services	61 (17.4)
	Manufacturing	29 (8.3)
	Financial industry	7 (2.0)
	Military and police education	47 (13.4)
	Unemployed	11 (3.1)
**Monthly income (NT $)**		
	<30,000	230 (65.7)
	30,001~50,000	94 (26.9)
	50,001~70,000	21 (6.0)
	>70,001	5 (1.5)
**Living area**		
	Northern Taiwan	236 (67.4)
	Central Taiwan	49 (14.0)
	Southern Taiwan	55 (15.7)
	Eastern Taiwan	7 (2.0)
	offshore islands	3 (0.9)
**Health status**		
	Excellent	28 (8.0)
	Good	171 (48.9)
	Normal	132 (37.7)
	Poor	19 (5.4)

### Importance-Performance Analysis

**Figure 2:**
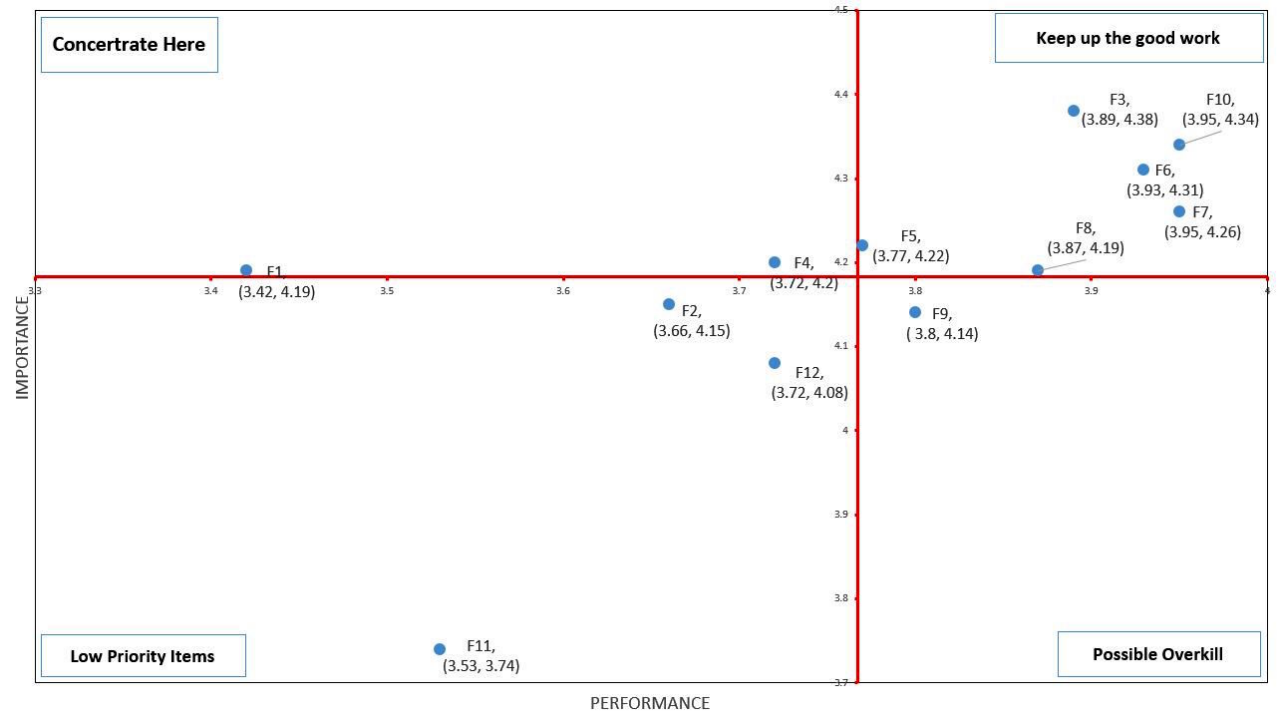
Quadrant diagram of the importance and performance of product establishment and functionality.

The Importance-performance Analysis (IPA) framework was introduced by Martilla and James [12]. It uses the mean of importance and satisfaction of performance of all items, and employs the intersection point as the origin to draw a quadrant diagram. As defined in quadrant 1 of Figure 2, it indicates high importance and high satisfaction of performance, which indicates that existing systems have strengths and should be maintained. Items in quadrant 2 have high importance but low satisfaction of performance. This quadrant is labeled as “Concentrate Here.” This category is labeled as “Keep up the good work.” In quadrant 3, low importance and low satisfaction of performance items renders it as “Low Priority.” Finally, quadrant 4 represents low importance and high satisfaction of performance, which suggests insignificant strengths and a possibility that the resources invested may be better used elsewhere.

In this study, the questionnaire was divided into “system functions” and “system design and security,” the two aspects that evaluate the relationship between importance and satisfaction of performance. We focused on the key area for improvement in the second quadrant of the IPA to identify the services showing high importance and low satisfaction of performance to determine the improvement priorities. This analysis is expected to provide direction for future enhancement and implementation of the personal EMR platform as a reference for government agencies and system developers.

In the system function part, as shown in Table 3, F7 (can maintain and keep complete personal health examination record) and F10 (can maintain and keep complete personal medical record) both have the highest satisfaction of performance. They are followed by F6 (can maintain and keep complete personal health record), F3 (can access PHR from other hospitals to avoid duplication of examinations, tests, and medication), and F8 (can maintain and keep complete personal medical image).The lowest satisfaction of performance is F1 (have detailed operating instructions). F3 has the highest importance, followed by F10 and F6.

**Table 3:** Satisfaction of performance and importance with the order of every question in system function part (order 1: most important or satisfied, 12: least important or satisfied).

Question	Satisfaction of performance, mean (SD)	Order	Importance, mean (SD)	Order	Quadrant	Chuchiming Index^b^
F1. Personal EMR^a^ platform have detailed operating instructions	3.42 (0.92)	12	4.19 (0.78)	7	Ⅱ	0.71
F2. Personal EMR platform lets patients integrate existing paper-based medical history	3.66 (0.83)	10	4.15 (0.73)	9	Ⅲ	0.11
F3. Personal EMR platform lets patients access their own PHR from other hospitals to avoid duplication of examinations, tests, and medication	3.89 (0.89)	4	4.38 (0.69)	1	Ⅰ	3.00
F4. Personal EMR platform lets patients integrate their own PHR data and provide continuous numerical statistics	3.72 (0.88)	8	4.20 (0.74)	6	Ⅱ	0.33
F5. Personal EMR platform allow doctors to add more details on the medical records	3.77 (0.92)	7	4.22 (0.77)	5	Ⅰ	0.40
F6. Personal EMR platform lets patients maintain and keep complete personal “health record” (such as disease history, medication history, and blood pressure)	3.93 (0.88)	3	4.31 (0.72)	3	Ⅰ	0.00
F7. Personal EMR platform lets patients maintain and keep complete personal “health examination record” (such as blood test and urine test reports)	3.95 (0.86)	1	4.26 (0.72)	4	Ⅰ	−0.75
F8. Personal EMR platform lets patients maintain and keep complete personal “medical image” (such as X-ray and MRI)	3.87 (0.86)	5	4.19 (0.76)	7	Ⅰ	−0.29
F9. Personal EMR platform lets patients maintain and keep complete personal “endoscopic image”	3.80 (0.89)	6	4.14 (0.78)	10	Ⅳ	−0.40
F10. Personal EMR platform lets patients maintain and keep complete personal medical record (such as diagnosis and prescriptions)	3.95 (0.86)	1	4.34 (0.73)	2	Ⅰ	−0.50
F11. Personal EMR platform lets patients share their PHR with family (friends) to enable them understand their health condition	3.53 (1.01)	11	3.74 (1.02)	12	Ⅲ	−0.08
F12. Personal EMR platform lets patients share their medical records with another physician as a reference when diagnosis is carried out	3.72 (0.91)	8	4.08 (0.84)	11	Ⅲ	−0.27

^a^EMR: electronic medical record.

^b^index developed by Dr Chuchiming. In this study, Chuchiming index>0 indicates items need concerted improvement (perceiving targets), Chuchiming index<0 indicates resources can be drawn from items (shifting resources), Chuchiming index=0 indicates items can fit people’s expectation (balancing items).

Figure 2 shows the quadrant distribution of the importance and satisfaction of performance of every system function of the personal EMR platform. The first quadrant (high importance and high satisfaction of performance) has F3, F5 (allows doctor to add more details on the medical records), F6, F7, F8, and F10. The items in the second quadrant (high importance and low satisfaction of performance) are F1 and F4 (can integrate their own PHR data and provide continuous numerical statistics). The items in the third quadrant (low importance and low satisfaction of performance) are F2 (existing paper-based medical history can be integrated), F11 (can be shared PHR with family), and F12 (can share PHR with physicians). The item in the fourth quadrant (low importance and high satisfaction of performance) is F9 (can maintain and keep complete personal endoscopic images).

In Figure 2, there are 4 functions with below average importance, including “sharing PHR with family and physician” (F11, F12), “integrating existing paper-based medical history” (F2), and “maintaining and keeping complete personal endoscopic image” (F9).

It also has 5 functions with below average satisfaction of performance, including “detailed personal EMR platform operating instructions” (F1), “integrating existing paper-based medical history” (F2), “sharing PHR with family or physician” (F11, F12), and “integrating their own PHR data and provide continuous numerical statistics” (F4).

It means a paperless process or sharing with others is not seen as important functions by the participants. They just want to have a platform that can let them maintain and keep complete personal records and basic medical images.

After conducting the Analysis of Variance (ANOVA) test, we didn’t find any significant difference between the various age groups, annual income groups, and health status groups, meaning that the results described above are consistent in every group.

In the system design and security part, as shown in Table 4, S2 (can let patients access their own PHR quickly) have highest satisfaction of performance and followed by S3 (lets patients login by multiple methods), S1 (interface should be simple and easy to understand) and S4 (can let patients access their own PHR under a secure environment). The lowest satisfaction of performance is S5 (let patients can login by id and password). S3 have highest importance, followed by S4 and S7 (can let patients set access right).

**Table 4:** Satisfaction of performance and importance with their order of every question in system design and security part (order 1: most important or satisfied, 7: least important or satisfied).

Question	Satisfaction of performance, mean (SD)	Order	Importance, mean (SD	Order	Quadrant	Chuchiming Index^c^
S1. The interface should be simple and easy to understand	3.82 (0.91)	3	4.28 (0.76)	4	Ⅳ	−0.25
S2. Personal EMR^a^ platform lets patients access their own PHR^b^ quickly	3.91 (0.86)	1	4.25 (0.71)	7	Ⅳ	−0.86
S3. Personal EMR platform lets patients login by multiple methods such as citizen digital certificate, id, and password.	3.87 (0.92)	2	4.39 (0.86)	1	Ⅰ	1.00
S4. Personal EMR platform lets patients access their own PHR under a secure environment	3.78 (0.96)	4	4.34 (0.76)	2	Ⅱ	1.00
S5. Personal EMR platform lets patients login by id and password	3.73 (0.85)	7	4.27 (0.82)	5	Ⅲ	0.40
S6. Personal EMR platform lets patients login by “Citizen Digital Certificate”	3.76 (0.95)	6	4.26 (0.80)	6	Ⅲ	0.00
S7. Personal EMR platform lets patients set access rights for every physician, family, or friend.	3.78 (0.92)	5	4.29 (0.76)	3	Ⅲ	0.67

^a^EMR: electronic medical record.

^b^PHR: personal health record.

^c^index developed by Dr Chuchiming. In this study, Chuchiming index>0 indicates items need concentrated improving (perceiving targets), Chuchiming index<0 indicates resources can be drawn from items (shifting resources), Chuchiming index=0 indicates items can fit people’s expectation (balancing items).

**Figure 3:**
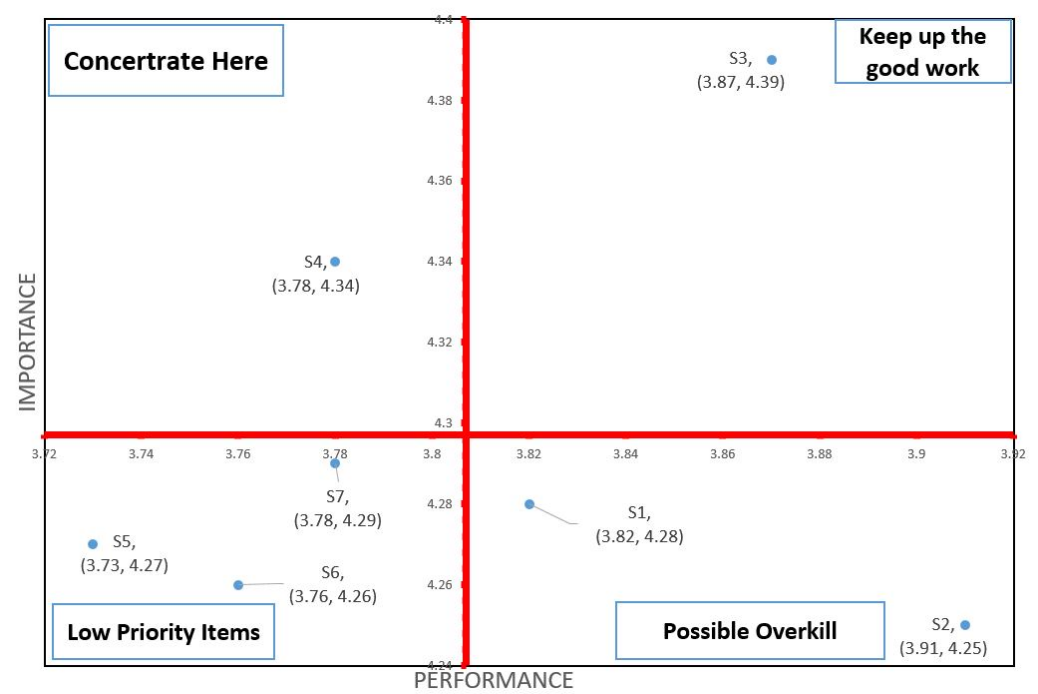
Quadrant diagram of the importance and performance of the security of the system.

Figure 3 shows the quadrant distribution of the importance and satisfaction with performance of the system design and security aspects of the Personal EMR Platform. The item in the first quadrant (high importance and high satisfaction with performance) is S3. The item in the second quadrant (high importance and low satisfaction with performance) is S4. The items in the third quadrant (low importance and low satisfaction with performance) are S5, S6 (lets patients log in using a citizen digital certificate) and S7. The fourth quadrant (low importance and high satisfaction with performance) includes S1 and S2.

In Figure 3, we also can find most of the system design and security below average in importance, besides letting patients log in by multiple methods (S3) and letting patients access their own PHR under a secure environment (S4); however, even the next personal EMR platform has been designed with multiple security protection methods. Participants still feel concerned about the system security issue; the satisfaction of performance of most questions is low, besides S1, S2, and S3. Thus, system security is still a big issue when building this kind of platform in the future.

After conducting the ANOVA test, we found some differences in system design and security part in terms of the importance attributed to system design and security part, as shown in Table 5. The mean importance of the age group of less than 20 years is lower than the values of other groups (*P*=.05). It means this group is not very concerned about the security issue. Also, in satisfaction with performance of system security (according to a group's monthly income and health status), the group of monthly income between NT $50K and NT $70K have lowest satisfaction with performance (*P*=.02). This was also found in the group which thinks they are poor health participants. (*P*=.002).

**Table 5:** Differences found in analysis of importance and satisfaction of performance between every demographic class in system design and security part by analysis of variance (ANOVA) test.

Attitude	Category		N	Mean (SD)	*F*	*P* value
Importance					
	**Age (years)**					
		<20	46	4.034 (0.657)	2.586	.05
		21-30	238	4.315 (0.637)		
		31-40	56	4.258 (0.616)		
		>40	10	4.371 (0.629)		
	**Highest education level**					
		High school	8	4.161(0.442)	0.504	.60
		University degree	228	4.251(0.640)		
		Institute or above	114	4.316(0.656)		
	**Employment**					
		Students	195	4.315(0.624)	0.734	.60
		Services	61	4.253(0.661)		
		Manufacturing	29	4.118(0.615)		
		Financial industry	7	4.122(0.550)		
		Military and police education	47	0.204(0.720)		
		Unemployed	11	4.351(0.611)		
	**Monthly income (NT $)**					
		<30,000	230	4.276(0.634)	0.270	.76
		30,001~50,000	94	4.280(0.615)		
		50,001~70,000	26	4.181(0.727)		
	**Living area**					
		Northern Taiwan	236	4.308(0.636)	1.510	.20
		Central Taiwan	49	4.262(0.629)		
		Southern Taiwan	55	4.171(0.648)		
		Eastern Taiwan	7	3.796(0.552)		
		offshore islands	3	4.333(1.033)		
	**Health status**					
		Poor	19	4..180(0.789)	0.349	.79
		Normal	132	4.260(0.664)		
		Good	171	4.272(0.615)		
		Excellent	28	4.367(0.594)		
Satisfaction of performance					
	**Age (years)**					
		<20	46	3.637(0.739)	1.533	.20
		21-30	238	3.867(0.773)		
		31-40	56	3.737(0.794)		
		>40	10	3.614(1.149)		
	**Highest education level**					
		High school	8	3.821(0.457)	1.097	.34
		University degree	228	3.853(0.766)		
		Institute or above	114	3.719(0.839)		
	**Employment**					
		Students	195	3.834(0.762)		
		Services	61	3.763(0.872)		
		Manufacturing	29	3.828(0.732)		
		Financial industry	7	3.918(0.605)		
		Military and police education	47	3.699(0.869)		
		Unemployed	11	3.948(0.652)		
	**Monthly income (NT $)**					
		<30,000	230	3.822(0.763)	4.074	.02
		30,001~50,000	94	3.888(0.786)		
		50,001~70,000	26	3.401(0.889		
	**Living area**					
		Northern Taiwan	236	3.810(0.733)	0.962	.43
		Central Taiwan	49	3.843(0.764)		
		Southern Taiwan	55	3.813(0.765)		
		Eastern Taiwan	7	3.306(0.871)		
		offshore islands	3	4.238(0.719)		
	**Health status**					
		Poor	19	3.143 (0.838)	5.077	.002
		Normal	132	3.874 (0.723)		
		Good	171	3.825 (0.774)		
		Excellent	28	3.852 (0.934)		

Table 6 shows the result of the ANOVA test of importance and satisfaction regarding performance between every demographic class in system functions. In this part, only the group with the monthly income between NT $50k and NT $70k, the mean satisfaction of performance, is lower than that of others (*P*=.01). This means income has a strong correlation to the satisfaction with performance of system functions.

**Table 6:** Differences found in analysis of importance and satisfaction with performance between every demographic class in system functions part by analysis of variance (ANOVA) test.

Attitude	Category		N	Mean (SD)	*F*	*P* value
Importance					
	**Age (years)**					
		<20	46	3.984(0.677)	2.406	.07
		21-30	238	4.209(0.588)		
		31-40	56	4.198(0.536)		
		>40	10	4.417(0.626)		
	**Highest education level**					
		High school	8	4.208(0.396)	1.204	.30
		University degree	228	4.148(0.607)		
		Institute or above	114	4.254(0.588)		
	**Employment**					
		Students	195	4.233(0.594)	1.565	.17
		Services	61	4.111(0.636)		
		Manufacturing	29	3.994(0.440)		
		Financial industry	7	3.845(0.667)		
		Military and police education	47	4.234(0.632)		
		Unemployed	11	4.205(0.503)		
	**Monthly income (NT $)**					
		<30,000	230	4.198(0.610)	0.387	.68
		30,001~50,000	94	4.230(0.560)		
		50,001~70,000	26	4.154(0.635)		
	**Living area**					
		Northern Taiwan	236	4.215(0.596)	1.037	.27
		Central Taiwan	49	4.197(0.528)		
		Southern Taiwan	55	4.089(0.594)		
		Eastern Taiwan	7	3.786(0.829)		
		offshore islands	3	4.111(1.197)		
	**Health status**					
		Poor	19	4.154(0.709)	0.267	.85
		Normal	132	4.152(0.605)		
		Good	171	4.204(0.594)		
		Excellent	28	4.232(0.521)		
Satisfaction of performance					
	**Age (years)**					
		<20	46	3.601(0.699)	1.282	.28
		21-30	238	3.815(0.717)		
		31-40	56	3.702(0.757)		
		>40	10	3.725(1.069)		
	**Highest education level**					
		High school	8	3.844(0.649)	0.522	.59
		University degree	228	3.792(0.713)		
		Institute or above	114	3.710(0.780)		
	**Employment**					
		Students	195	3.788(0.710)	0.734	.60
		Services	61	3.745(0.820)		
		Manufacturing	29	3.690(0.600)		
		Financial industry	7	3.738(0.598)		
		Military and police education	47	3.722(0.845)		
		Unemployed	11	3.924(0.619)		
	**Monthly income (NT $)**					
		<30,000	230	3.747(0.702)	4.366	.01
		30,001~50,000	94	3.904(0.754)		
		50,001~70,000	26	3.442(0.831)		
	**Living area**					
		Northern Taiwan	236	3.757(0.765)	0.526	.72
		Central Taiwan	49	3.847(0.610)		
		Southern Taiwan	55	3.759(0.679)		
		Eastern Taiwan	7	3.476(0.830)		
		offshore islands	3	4.208(1.018)		
	**Health status**					
		Poor	19	4.180(0.789)	0.349	.79
		Normal	132	4.260(0.664)		
		Good	171	4.272(0.615)		
		Excellent	28	4.367(0.594)		

## Discussion

### Principal Findings

Liang’s [14] awareness, want, and adoption gap ratio (AWAG) segment matrix that analyzes the digital divide in eHealth services divides into four groups: strong opened group, generic opened group, generic closed group, and strong closed group by the participants’ awareness and want rate. Results showed that for both eHealth services, the digital divides of awareness, want, and adoption existed across demographic variables, as well as between computer owners and nonowners, and between Internet users and nonusers. It means users' attitudes and experience are the most important factors determining whether and how eHealth services will be used.

This study focused on system functions and system design and security to identify the key functions of patients’ views by using IPA to determine which functions people think are important for them. The study can serve as a guide when this kind of platform is built in the future. We found people do not need overly complex functions; they just want a platform that lets them integrate and manage their medical visits, health examination, and life behavior record. However, viewing the Web-based medical image is not an important function for them, and they also do not care if their PHR is shared with others. Surprisingly, most of the participants think the system security design is not important. The importance of 5 of 7 questions in total is below average (4.29, 1: most not important, 5: most important); they only think “letting patients login by multiple methods, such as citizen digital certificate, id and password” and “letting patients access their own PHR under a secure environment” is important. Satisfaction in performance is also not high. The significance of only 3 of 7 questions is higher than average (3.8). We also found that the satisfaction regarding performance is of lower importance not only in system functions aspects but also in system design and security aspects. This means that although they think some of the system functions and system security design are not very important, they are not satisfied even when the system has these functions. However, although future personal EMR platform could have many system security designs, people may still not think these are enough to make sure the system is secure.

With increased acceptance of PHR, users increasingly believe that the use of a personalized EMR could help them understand their own medical records. Research on PHR became more popular in recent years; however, most of the research was focused on investigating the use of doctors and nurses’ satisfaction. Rarely were the patients’ views given consideration. Some studies have also pointed out that if the patients are satisfied with the use of information in this context, one could improve the feasibility of using PHR [15]. Some surveys have indicated that consumers want Web-based access to their PHR [16,17]; however, they still have some doubts, including the possibility that full and open access to personal medical information could bring up privacy concerns [18]; the problems of usability and security issues, complexity in the use of PHRs [2]; and perceived usefulness, motivation, patient and health professional interaction, lack of time and workload, resources availability, management, outcome expectancy, and interoperability [19]. Agarwal et al [20] found provider satisfaction, interactions between environmental factors (communication tactics and value of the tool functionality), and interactions between patient activation and tool empowerment potential were significantly associated with behavioral intentions to use the PHR. Patients who believed the tool to be empowering demonstrated greater intention to use it, which was further enhanced for highly motivated patients. Baird [21] and Liu [22] also thinks that the concerns and challenges of using PHR are more focused on discussions regarding confidentiality, integrity, authorization, access control, portability, efficiency, scalability of solutions, and issues related to user experiences.

Compared with these studies, our results are similar; however, our study focused on system functions and system design and security. These questions are related to perceived usefulness, patient and health professional interaction, and management and interoperability aspects for perceived usefulness. Our research found participants pay more attention to how PHR can help them manage their health records, such as medical records, health examination records, and medical images, while also helping them avoid duplication of examinations, tests, and medication. For patient and health professional interactions, participants think PHR can let physicians add more details to the medical records; regarding management and interoperability, it was found in our study that detailed operating instructions, log in methods, and operation with secure methods are important for the participants, but the satisfaction with performance are below average.

Overall, the issues receiving the most user attention were the system functions, circulation, integrity, ease of use, continuity of the PHR, and data security and privacy protection. “My health bank” query service implemented by the government in recent years allows patients to check medical records (including the date of medical treatment, drug use, inspection report, and the doctor's advice) for 1 year through a personal certificate. Through this access, the integrity and continuity of PHRs can be achieved, but the propaganda for publicity still needs to be strengthened. In future, the government should provide functions and services that can meet the needs of the users, which will also enable the users to understand their own medical records, enhance understanding of the disease for the doctors, enhance the quality of EMR writing, reduce duplicated examinations, and develop holistic care.

### Limitations

The personal EMR platform concept proposed in this study is relatively new in Taiwan. Most people are not yet aware of this process. Therefore, their understanding of the personal EMR platform may be poor. In this study, the system was introduced by way of a video to ensure that the participants understood as much as possible before they began to fill out the Web-based questionnaire, which we expected to reduce possible errors. Although the accuracy and validity of the data were not validated due to the lack of sample representation and extrapolation of the results, this prospective study can act as a reference for future studies on the development of the domestic personal EMR platform.

This study was conducted only by a Web-based questionnaire survey. Most of the participants were people with high Internet usage, young, and the areas of residence were concentrated in the north, which could cause the findings to be generalized to the whole population. Therefore, if the results were to extend to other regions or remote areas, there may be a gap. We suggest that future researches could use diversification methods for the survey.

## Abbreviations

EMR: electronic medical records
PHR: Personal Health Records
