# Metadata Correction: Importance-Performance Analysis of Personal Health Records in Taiwan: A Web-Based Survey

**DOI:** 10.2196/jmir.7944

**Published:** 2017-06-09

**Authors:** Hsiao-Hsien Rau, Yi-Syuan Wu, Chi-Ming Chu, Fu-Chung Wang, Min-Huei Hsu, Chi-Wen Chang, Kang-Hua Chen, Yen-Liang Lee, Senyeong Kao, Yu-Lung Chiu, Hsyien-Chia Wen, Anis Fuad, Chien-Yeh Hsu, Hung-Wen Chiu

**Affiliations:** ^1^ Graduate Institute of Biomedical Informatics Taipei Medical University Taipei Taiwan; ^2^ Administration Department Healthconn Corp Taipei Taiwan; ^3^ Institute of Medical Informatics Department of Computer Science of Information Engineering National Cheng Kung University Tainan Taiwan; ^4^ School Of Public Health National Defense Medical Center Taiepi Taiwan; ^5^ National Health Insurance Administration Ministry of Health and Welfare Taiepi Taiwan; ^6^ School of Nursing College of Medicine Chang Gung University Linkou Taiwan; ^7^ Division of Endocrinology, Department of Pediatrics Linkou Chang Gung Memorial Hospital Linkou Taiwan; ^8^ Internet of Things Laboratory Chunghwa Telecom Laboratories TaoYuen Taiwan; ^9^ School of Health Care Administration Taipei Medical University Taipei Taiwan; ^10^ Department of Biostatistics Epidemiology and Population Health, Faculty of Medicine Universitas Gadjah Mada Yogyakarta Indonesia; ^11^ Department of Information Management National Taipei University of Nursing and Health Sciences Taipei Taiwan; ^12^ Master Program in Global Health and Development Taipei Medical University Taipei Taiwan

The authors of “Importance-Performance Analysis of Personal Health Records in Taiwan: A Web-Based Survey” (J Med Internet Res 2017;19(4):e131) overlooked omissions in the metadata. Two of the article's authors should have been designated as having made an equal contribution. The authors are Dr. Yu-Lung Chiu and Dr. Hsyien-Chia Wen. Their contributions to this paper were significant, and we apologize for the omission in the original article.

This correction has been made in the online version of the paper on the JMIR website on June 9, 2017, together with the publication of this corrigendum.

A correction notice has been sent to PubMed, and the publication was resubmitted to Pubmed Central and other full-text repositories.

